# Impact of Perceived Dorsiflexion and Plantarflexion in the Squat and Countermovement Jumps

**DOI:** 10.3390/muscles5010005

**Published:** 2026-01-12

**Authors:** Flávio Ventura, Filipe Maia, Ricardo Maia Ferreira, Nuno Pimenta, Ricardo Pimenta

**Affiliations:** 1 Polytechnic Institute of Maia N2i, Social Sciences, Education and Sport School, Avenida Carlos de Oliveira Campos, Castêlo da Maia, 4475-690 Maia, Portugal; a036787@ipmaia.pt (F.V.); fm.filipemaia@gmail.com (F.M.);; 2 Research Center in Sports Science, Health Science and Human Development (CIDESD), University of Maia, 4475-690 Maia, Portugal; 3 Sport Physical Activity and Health Research & Innovation Center (SPRINT), 4960-320 Melgaco, Portugal; 4 Scientific-Pedagogical Unit of Physiotherapy, Coimbra Health School, Polytechnic Institute of Coimbra, Rua 5 de Outubro, S. Martinho do Bispo, 3045-043 Coimbra, Portugal; 5 Department of Rehabilitation and Optimization of Performance (DROP), Futebol Clube de Famalicão—Futebol SAD, Rua da Academia F.C. Famalicão 300, Esmeriz, 4760-482 Famalicão, Portugal; 6 Porto Biomechanics Laboratory, Faculty of Sport, University of Porto, 4150-564 Porto, Portugal

**Keywords:** vertical jump, dorsiflexion, plantar flexion, squat jump, countermovement jump

## Abstract

Muscular strength plays a crucial role in sports performance and is often evaluated using vertical jump tests such as the Squat Jump (SJ) and Countermovement Jump (CMJ). Measurements based on flight time (FT) assume that takeoff and landing postures are identical, yet differences in ankle position can introduce systematic errors. This study examined whether dorsiflexion (DF) or plantarflexion (PF) of the ankle during the flight phase affects jump height. Forty-three active university students completed four repetitions each of SJ and CMJ under DF and PF across two sessions. Jump heights were recorded using a Chronojump-Boscosystem platform. No significant difference was observed in SJ between DF and PF, while CMJ heights were consistently higher under DF (DF: 28.29 cm ± 7.7 cm vs. PF: 27.08 cm ± 7.03 cm, *p* = 0.001; d = 0.16). Notably, the effect of DF appeared more pronounced in CMJ, suggesting that higher jumps are more sensitive to postural variations. These findings could suggest that DF can artificially increase jump heights as measured on a jump platform, without reflecting true improvements in force production. Coaches and practitioners should interpret FT-derived data with caution, particularly for higher jumps. Future research combining precise motion capture with force platforms could directly track center-of-mass changes and validate this mechanism.

## 1. Introduction

Muscular strength is a key physical quality in sports performance, influencing movements such as jumps, sprints, and changes of direction in various sports, such as football, basketball, and athletics [1]. In many sports, monitoring fatigue recurring to neuromuscular tests before training sessions is a common practice such as the Isometric Mid-Thigh Pull (IMTP) and the Countermovement Jump (CMJ), to assess athletes’ recovery and readiness [2,3]. For example, Pimenta et al. used the IMTP to examine post-match neuromuscular fatigue in elite youth soccer players, highlighting its usefulness in tracking recovery [2]. Similarly, Tito et al. analyzed CMJ performance to understand the evolution of fatigue after official matches, providing valuable insights into the temporal dynamics of recovery [3]. Indeed, vertical jump tests, such as the CMJ and the Squat Jump (SJ), are widely used in sports to assess lower-limb explosive strength and neuromuscular efficiency, to profile physical performance, measure fatigue and recovery, and guide return-to-play decisions [1,4]. Previous research consistently shows that CMJ results in greater jump heights compared to squat jumps (SJ) [5,6,7]. This can be explained by the fact that CMJ includes a stretch-shortening cycle (SSC), which enhances jump performance by using elastic energy and potentiation from muscle pre-activation [5,7]. On other hand, the SJ isolates concentric force production by eliminating the SSC effects, offering greater insight into muscular strength [8].

To achieve kinematic details (joint angles, segmental velocities and the trajectory of the center of mass (CoM) throughout the movement), researchers often combine motion capture systems with force platforms. More recently, alternative motion capture approaches have emerged, including wearable photonic sensing systems that integrate stretchable elastomer optical fibers for precise motion detection and remote monitoring. For example, Guo et al. developed ultra-stretchable chromotropic fibers for 3D motion capture integrated into textiles, capable of tracking joint trajectories in real time [9]. While these innovative wearable systems show great potential for movement analysis, force platforms are considered the gold standard for jump assessment [10,11,12], with jump height being typically calculated from take-off velocity derived from vertical force data (using impulse–momentum approach), although flight time (FT) is also frequently used for comparison with other measurement systems such as contact mats or video analysis [10,11,12]. This combination of motion capture systems and force platforms provides precise data, painting a full picture of neuromuscular performance [13]. Moreover, it has been reported that force platforms are reliable under standardized conditions, and relies on the assumption that takeoff and landing postures are identical [14]. Nevertheless, the significant investment required for force platforms has led to the popularity of alternative devices such as jump platforms, optical systems, and smartphone apps in sports performance and rehabilitation environments [15].

Among these, jump platforms have been highlighted as a practical and valid alternative for assessing jump performance, providing reliable estimates of jump height through FT-based calculations [16]. In both methodologies (force or jump platforms) athletes frequently land with greater joint flexion (particularly at the ankle) which results in a lower CoM at landing (ground contact). This alteration bias the flight time, leading to overestimated jump height [14]. Indeed, a biomechanical simulation by Gonçalves et al. (2024) demonstrated how differences in ankle dorsiflexion (DF) at landing versus takeoff can misrepresent FT-based jump height estimations, which could lead to overestimations of up to 60% [17]. Therefore, these results emphasized the need for careful interpretation of FT-derived data, and adequate technique education to athletes. However, Gonçalves et al. did not analyze whether the athlete was in DF or plantar flexion (PF) during the flight phase, but rather focused only on toe-off and landing [17].

Compared to a PF condition, DF during the flight phase, especially during the moments previous to landing would theoretically contribute to a longer FT as the foot would take longer to contact the ground, subsequently biasing towards higher jump heights outputs from the FT method. Therefore, it is possible that, despite the same take-off and landing positions on the platform, alterations occurring during the jump may lead to differences in flight time and, consequently, in the estimation of jump height. Consequently, if athletes adopt strategies that modify their flight time (DF), this could lead to errors in jump height estimation, which in turn would impact assessments of explosive strength performance [18,19] and even neuromuscular fatigue management [3]. Although some practitioners assume this error is consistent across trials, the magnitude of the misrepresentation undermines the validity of absolute jump height measures using FT [20]. Ensuring identical posture at takeoff and landing is practically difficult, making these measures vulnerable to systematic error [17] which could also be impacted by the ankle position during the jump. It should be noted that kinematic analysis is often unavailable in field settings due to its high cost [21] and the technical requirements necessary to ensure high-quality image capture, such as sufficient frame rate to capture fast movements without motion blur [22], adequate number and optimal placement of cameras to avoid occlusion and ensure spatial coverage [23], strong and consistent lighting with suitable shutter speed and lens focus to maintain image sharpness [24], and rigorous system calibration and synchronization (including camera calibration, lens distortion correction, and alignment with any force plates) [25]. Therefore, the use of familiarization sessions to enhance participants’ perception of ankle positioning, may help athletes perform the task more consistently, thus allowing a reliable comparison between DF and PF positions during the jump. By standardizing the adequate technique during the flight phase and concomitantly explaining the deleterious effects induced by the inadequate technique performance in vertical jump testing, practitioners would be objectively informed on the importance of applying rigorous test execution criteria to obtain accurate outputs that will ultimately contribute to the comprehension of performance and fatigue monitoring derived from vertical jump testing. Although previous research has examined differences between jump types and technical factors influencing flight time, limited evidence exists on how self-perceived ankle positioning during the flight phase may bias jump height estimations derived from FT. Establishing this relationship is particularly relevant for ensuring the comparability of jump assessments performed longitudinally, where variations in technique or ankle positioning could compromise data consistency. Therefore, this study aims to address this methodological gap by providing new insights through a practical design, without the need for additional equipment, analyzing the effects of ankle positioning perception on jump height calculations, controlled via visual feedback from a strength and conditioning coach.

The present study aims (i) to compare vertical jump height between SJ and CMJ, and (ii) to examine how the self-perceived DF and PF during the flight phase influence vertical jump height in SJ and CMJ using the FT method. We hypothesize that (i) CMJ will be greater than SJ height; and (ii) jumps performed with DF perception will yield higher values compared to those performed with PF perception.

## 2. Results

**Figure 1:**
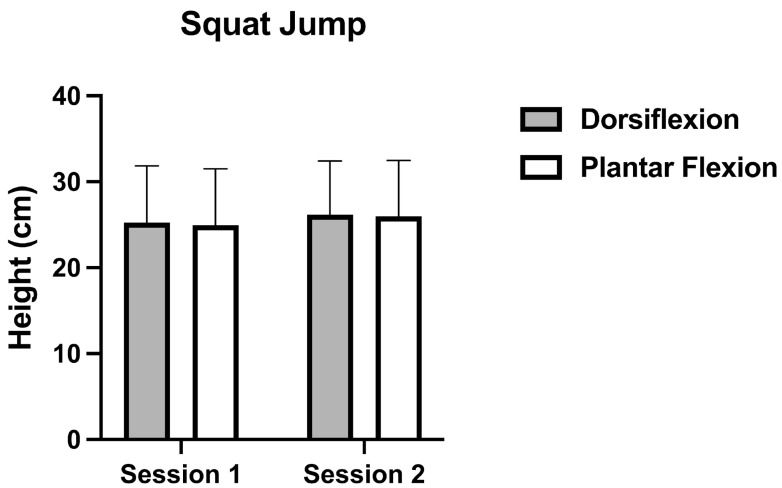
Squat jump height in Session 1 and 2 with DF (gray) and PF (white) conditions.

Regarding the comparison between jump type (CMJ vs. SJ), significant differences were observed (*p* = 0.001; d = 0.29) with CMJ reporting higher heights. For SJ (Figure 1), a non-significant effect was observed between ankle (*p* = 0.618; η^2^p = 0.006). Specifically, no differences were observed between DF and PF (DF: 25.72 cm ± 6.39 cm vs. PF: 25.46 cm ± 6.52 cm; *p* = 0.618; d = 0.04).

**Figure 2:**
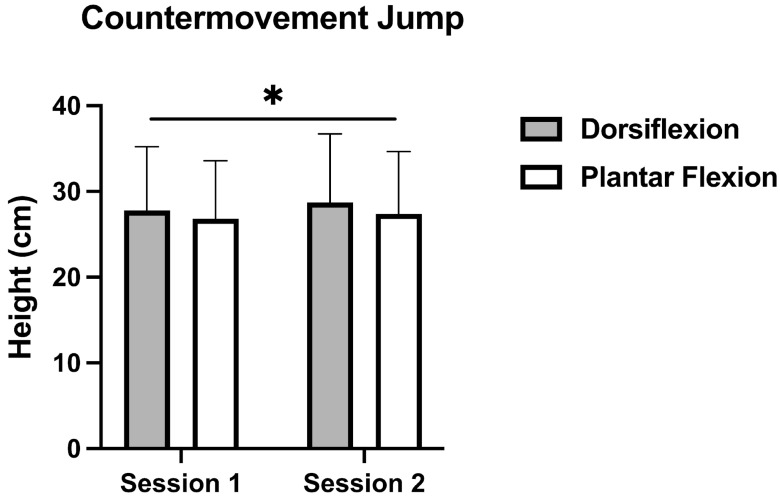
Countermovement jump height in Session 1 and 2 with DF (gray) and PF (white) conditions. * *p* < 0.05.

On the other hand, a statistically significant effect was observed for CMJ (Figure 2) regarding ankle position (*p* = 0.001; η^2^p = 0.216). Particularly, DF achieved greater jumping heights in comparison to PF (DF: 28.29 cm ± 7.7 cm vs. PF: 27.08 cm ± 7.03 cm, *p* = 0.001; d = 0.16).

In SJ, small percentage changes were observed between DF and PF conditions (1.27% in Session 1 and 0.76% in Session 2), suggesting that SJ height remained highly consistent regardless of ankle position. In the CMJ, slightly greater percentage differences were observed between DF and PF conditions, with values of 3.7% in Session 1 and 4.8% in Session 2.

## 3. Discussion

This study aimed to determine whether instructed ankle position during the flight phase (DF vs. PF) affects vertical jump height derived from FT in the SJ and CMJ. In accordance with our first hypothesis, the CMJ produced higher jump heights than the SJ. Indeed, participants achieved an average CMJ height of 28.29 ± 7.70 cm (DF) and 27.08 ± 7.03 cm (PF), whereas SJ heights were lower (DF: 25.72 ± 6.39 cm; PF: 25.46 ± 6.52 cm). The CMJ exceeded the SJ (*p* = 0.001; d = 0.29), by approximately 10.2%, which aligns with previous findings reporting differences between 5 and 15% depending on population and sex [5,6,7]. For example, Bobbert et al. [5] observed an 8–12% advantage of CMJ over SJ in recreationally trained males, whereas Donahue et al. [6] reported a ~10% difference in a similar population. Moreover, Kozinc et al. [7] further demonstrated that the magnitude of CMJ–SJ differences varies with sex and sport background, with male athletes and those engaged in explosive sports exhibiting larger disparities, while females and participants with lower training experience showed smaller ones. In particular, Kozinc et al. [7] highlighted that vertical jump performance is strongly modulated by both sex and sport background, reporting a significant gender and sport interaction alongside main effects of gender and sport. Across almost all sports analyzed, male athletes displayed significantly higher CMJ heights than female athletes, reflecting greater neuromuscular capacity and stretch–shortening cycle efficiency. Short-distance runners achieved the highest CMJ heights overall, followed by volleyball and soccer players, whereas distance runners, dancers and tennis players exhibited the lowest values. It should be noted that, physical education students (same sample of this study) occupied an intermediate position: their CMJ performance was significantly higher than that of tennis players but lower than that of soccer or volleyball players. However, in the present study the values were lower than the previous study [7]. This pattern suggests that individuals with general but non-specialized training backgrounds (such as the participants in our study) tend to produce moderate jump heights compared to elite athletes, which may partly explain the magnitude of the CMJ–SJ differences observed. These variations collectively highlight the influence of neuromuscular capacity, training status and SSC efficiency on vertical jump performance. The close agreement between our results and the literature reinforces the notion that the SSC consistently contributes to enhanced CMJ performance [8,26].

Regarding our second hypothesis, differences between DF and PF were found in the CMJ, supporting previous findings that DF at landing can lower the center of mass and artificially extend flight time, causing an overestimation of jump height in FT based systems [17]. However, in the present study, dorsiflexion was maintained only during the flight phase and not during landing, which may explain the smaller magnitude of the observed effect. In the SJ, however, no differences were observed. One possible explanation could be the shorter flight times associated with lower jump heights [26], which leave less time for athletes to perceive and adjust their ankle position during the jump. Therefore, it could be suggested that shorter flight time minimizes the risk of overestimation. Moreover, the relatively low SJ heights in our participants may have also further limited the opportunity for postural adjustments, explaining the lack of statistical differences. Although no differences were observed between DF and PF conditions in SJ, this result should be interpreted with caution, as the absence of videographic analysis limits our ability to confirm whether participants truly adopted the instructed ankle positions.

The pattern of results suggests that the longer flight durations associated with the CMJ amplify the influence of ankle position on FT-derived jump height. In the present study, a mean difference of approximately 4% between PF and DF was noted for the CMJ at an average jump height of 28 cm. Although Gonçalves et al. (2024) did not analyze DF or PF during the flight phase, their simulation of different toe-off and landing scenarios indicated that extreme variations in ankle position could lead to overestimations of up to 60%, being influenced of a landing phase in DF with knee flexion [17]. In the present study, feedback was provided to ensure that participants consistently maintained full knee extension throughout the jump, both during familiarization sessions and testing sessions, which could be the of small differences (max. 5%) between PF and DF, suggesting that standardized instructions to keep the knee extended during testing may help mitigate such overestimations. Based on previous literature [27], it is reasonable to speculate that populations capable of higher jumps (35–40 cm) could display even greater discrepancies, as the extended FT increases the potential for measurement error.

The difference of approximately 4% in CMJ height between DF and PF, although statistically significant, corresponds to a small effect size (*d =* 0.16). From a practical standpoint, such a magnitude is unlikely to reflect meaningful changes in neuromuscular performance or to influence competitive outcomes. However, in applied monitoring contexts (where small differences in jump height are often used to guide training load adjustments or return-to-play decisions) this level of variation could lead to misinterpretation if ankle position is not standardized. Therefore, even small errors like the one observed here warrant careful consideration to ensure valid longitudinal tracking. These findings have important implications for sports science and applied practice. First, practitioners using FT-based systems such as contact mats or force platforms should consider standardizing ankle position instructions to reduce systematic error in jump height estimates. Incorporating familiarization trials, verbal cues, and proprioceptive training may help athletes adopt more consistent ankle positions during the flight phase [15,20]. Such measures can enhance within-athlete reliability and improve the validity of longitudinal monitoring programs, including the assessment of training adaptations, fatigue management, and return-to-play readiness [1,4,20]. Moreover, when testing athletes with higher jump capacities, practitioners should interpret FT-derived results with caution, as the magnitude of potential overestimation may be proportionally greater [17,27]. This issue is particularly relevant, as minor postural variations can produce measurable differences in jump height. This is particularly relevant in applied contexts such as monitoring training adaptations, fatigue management, or return-to-play decisions, where small differences in jump height can influence key choices. Moreover, as athletes achieve higher jumps, the influence of dorsiflexion appears to become more pronounced, making consistent technique control essential for well-trained or elite populations. It should be noted that consistently applying the same criterion (DF or PF) over time may partially mitigate this effect. Nevertheless, comparisons between athletes and across studies remain limited, and the potential impact of daily variations in DF angles on jump height has yet to be fully determined. Future studies should integrate high-precision motion capture systems with synchronized force platforms and have participants with more jump capacity to examine the effect of PF versus DF and to assess whether athletes’ perception of ankle joint angles remains consistent across both conditions.

Validation studies of flight-time-based systems [14,17,20] have consistently shown that technical or postural inconsistencies may lead to artificial alterations in jump height, which could be erroneously interpreted as genuine performance improvements. Therefore, if such variations are not accounted for as potential sources of measurement error, there is a significant risk that training decisions or return-to-play protocols could be guided by methodological artifacts rather than true neuromuscular adaptations. Additionally, an alternative to the FT method (particularly when landing technique may vary across trials) is the use of the impulse–momentum approach based on force plate measurements, which estimates jump height using the take-off velocity. However, this method is limited by the requirement of force plates, which are much more expensive and less practical than a contact mat, as well as by the need for a clear and stable baseline, free of oscillations, to avoid affecting the accuracy of the impulse–momentum calculation.

This study has some limitations. Firstly, no kinematic analysis was performed to ensure ankle position in both jumps, which means that we relied solely on participants’ perception. However, the aim of the study was to provide a fast and ecological approach to examine whether athletes’ perception influenced jump height. Secondly, although not a major limitation, the sample consisted of healthy individuals rather than trained athletes, which could result in smaller differences due to the reduced force production capacity. Therefore, it should not be generalized to other types of populations. Future research should include athletes with greater force production, synchronized force plates and incorporate kinematic measurements to more precisely assess the influence of ankle position across jump types.

## 4. Materials and Methods

### 4.1. Participants

A convenient sample size of forty-three active healthy university students (age: 21.3 ± 2.32 years; height: 1.75 ± 0.05 m; weight: 73.4 ± 11.07 kg; age range: 18 to 25 years; male: 30; female: 13) participated in this study. All individuals were informed of the experimental risks associated with the protocol and provided written informed consent prior to participation. To meet inclusion criteria, participants were required to be higher education students, and physically active according to the World Health Organization [28] (i.e., engaging in physical activity more than twice per week and accumulating at least 150 min of moderate-intensity activity, 75 min of vigorous-intensity activity, or an equivalent combination of both per week). All participants were excluded if they met any of the previously mentioned criteria or presented at least one of the following conditions: (i) a history of serious knee or hip injuries within the previous year (e.g., anterior cruciate ligament rupture, medial collateral ligament injury, femoroacetabular impingement, or groin injuries requiring surgical intervention, as well as any other severe musculoskeletal injuries likely to compromise sprint performance, maximal voluntary isometric contractions [MVIC], or shear wave elastography measurements); (ii) a history of low back disorders or current symptoms in the lumbosacral region; and (iii) the presence of electronic implants or ferromagnetic foreign bodies located in proximity to the thigh region [29].

Sample size was calculated a priori for the statistical test repeated measures analysis of variance (ANOVA; within factors), to detect a moderate difference (Effect size f = 0.25) in the variables under analysis, with an α value of 0.05 and 95% of statistical power [6] (G*Power, version 3.1.9.7), yielding a total sample of 36 participants. The effect size for sample calculation was based on the study of Donahue et al. [6]. However, a higher power was used to increase the probability of detecting true differences between conditions. All participants read and signed the informed consent form prior to the study, in accordance with the Declaration of Helsinki. This study was approved by the Ethics Committee of the Research Centre of the Polytechnic Institute of Maia (#01/2025).

### 4.2. Procedures

Before the data collection, participants performed a familiarization session with maximal and submaximal efforts to get used to the jump types (6 repetitions for each condition (DF and PF) and jumps (SJ and CMJ). After that, in two separated sessions, the participants performed four repetitions of the CMJ and four repetitions of the SJ: two with maximal DF and two with PF during the flight phase. The session started with a standardized warm-up protocol previously reported [30]. The sequence of jump conditions was randomized, and a two-minute rest interval was maintained between attempts.

For the CMJ, participants performed a rapid downward movement until approximately 90° of knee flexion, followed immediately by a maximal vertical jump in one continuous motion. For the SJ, participants flexed their knees to approximately 90° and held the position before jumping vertically as high as possible in response to an acoustic signal, strictly avoiding any countermovement. The range of the downward phase of the jump and the ankle position were performed according to the participants’ own perception.

**Figure 3:**
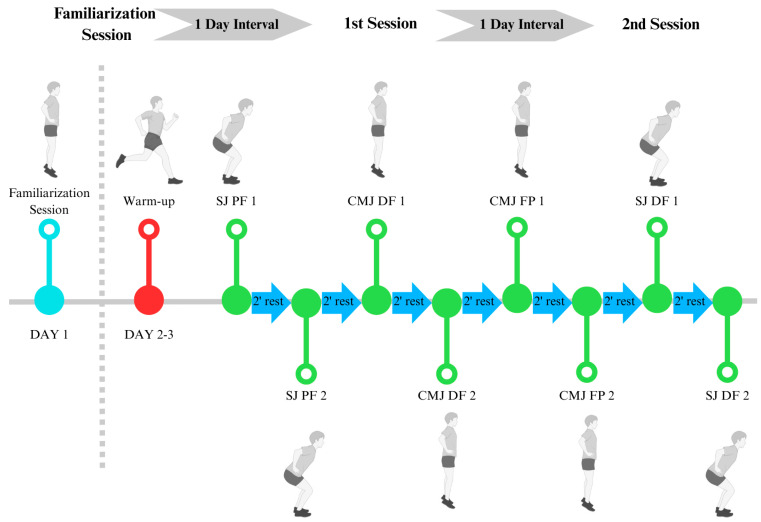
Example of timeline for the first and second data collection sessions. SJ, Squat Jump; CMJ, Countermovement Jump; PF, Plantar Flexion; DF, Dorsiflexion. Colors indicate the different experimental phases: light blue represents the familiarization session, red indicates the standardized warm-up, green denotes the jump test trials, and blue arrows represent 2-min rest intervals. The dashed vertical line indicates the transition between the familiarization period and the experimental sessions.

Participants were instructed to place their hands on their hips and maintain their arms steady throughout all jumps, following standard recommendations for vertical jump assessments [31,32,33]. Additionally, participants were explicitly instructed either to perform the jump with PF (for PF condition) throughout all phases of the movement or to reach maximal active dorsiflexion during the flight phase, in DF condition. In all trials, participants were required to land in a plantar-flexed position changing only the ankle position in the flight phase. The ankle position (DF or PF), jump technique, and jump height were monitored visually by a trained researcher which ensures that participants landing with the knee fully extended. Any jump that did not meet the protocol criteria was discarded, in order to ensure that only valid trials were included in the analysis. These procedures are detailed in Figure 3.

### 4.3. Instrumentation

The primary assessment tool was the jump platform system (Chronojump-Boscosystem, Barcelona, Spain). The jump platform system is an open-source hardware and software model (Chronojump 2.3.0-83). The platform is connected to a computer, which computes and stores FT with a temporal resolution of 1 ms. This system has been validated and shown to provide reliable measurements of flight time and jump height when compared with force platforms [34]. The jump height was calculated using the following equation: $Jump\;Height={\left(FT\right)}^{2}\times g/8$ [35], where g corresponds to the gravity acceleration (9.8 m/s^2^).

### 4.4. Statistical Analysis

All statistical analyses were conducted using IBM SPSS Statistics version 29.0 (IBM Corporation, Armonk, NY, USA), with statistical significance being set at *p* < 0.05. Prior to analysis, data were screened for normality using the Shapiro–Wilk test, confirming the assumption of normal distribution for all dependent variables. To assess differences in jump height across ankle positions and sessions, repeated measures ANOVA was computed with Ankle Position (dorsiflexion vs. plantarflexion), Session (Session 1 vs. Session 2) and Jump Type (CMJ vs. SJ) as factors. When significant main effects or interactions were identified, post hoc comparisons were carried out using the Bonferroni correction.

Additionally, the percentage change for jump height between ankle positions was calculated for each session using the following formula:

% of Change = (Jump height with Dorsiflexion − Jump height with Plantar flexion)/(Jump height with Dorsiflexion) × 100%

Effect sizes were reported using partial eta squared (η^2^p) for the ANOVA and interpreted as follows: small (0.01–0.05), medium (0.06–0.13), and large (>0.13) [36]. For pairwise comparisons, effect sizes were computed as Cohen’s d, and interpreted as small (0.20 to 0.49), moderate (0.50 to 0.79) and large (>0.80) [37].

## 5. Conclusions

The results of the present study may suggest that the ankle position during the flight phase can influence the vertical jump height measurements obtained from a jump platform system, particularly for the CMJ. Specifically, DF in flight appears to lead to higher values of CMJ height, while no effect was observed for the SJ. This effect could be explained due to the higher height values achieved on CMJ compared to SJ.

These results highlight how important it is to use caution when interpreting FT based jump height data. From a practical standpoint, coaches and sports scientists should be aware that apparent improvements in CMJ and SJ performance under dorsiflexion may not reflect true neuromuscular gains, but rather a measurement artifact.

## Data Availability

Data can be assessed by emailing the corresponding author.

## References

[1] Suchomel T.J., Nimphius S., Stone M.H. (2016). The Importance of Muscular Strength in Athletic Performance. Sports Med..

[2] Pimenta R., Cunha L., Nakamura F.Y. (2025). Impact of Post-Match Fatigue on Peak Force in Elite Youth Soccer Players: Analysis of 48 to 72 Hours Post-Match Using the Isometric Mid-Thigh Pull Exercise. Biol. Sport.

[3] Tito S., Maia F., Correia M., Ribeiro J. (2025). Recovery Patterns of Low-Frequency Fatigue in Elite Youth Soccer Players after Official Matches. Int. J. Sports Med..

[4] Maffiuletti N.A., Aagaard P., Blazevich A.J., Folland J., Tillin N., Duchateau J. (2016). Rate of Force Development: Physiological and Methodological Considerations. Eur. J. Appl. Physiol..

[5] Bobbert M.F., Gerritsen K.G., Litjens M.C., Van Soest A.J. (1996). Why Is Countermovement Jump Height Greater than Squat Jump Height?. Med. Sci. Sports Exerc..

[6] Donahue P.T., Wilson S.J., Williams C.C., Hill C.M., Garner J.C. (2021). Comparison of Countermovement and Squat Jumps Performance in Recreationally Trained Males. Int. J. Exerc. Sci..

[7] Kozinc Ž., Žitnik J., Smajla D., Šarabon N. (2022). The Difference between Squat Jump and Countermovement Jump in 770 Male and Female Participants from Different Sports. Eur. J. Sport Sci..

[8] Markovic G., Dizdar D., Jukic I., Cardinale M. (2004). Reliability and Factorial Validity of Squat and Countermovement Jump Tests. J. Strength Cond. Res..

[9] Guo Y., Guo Y., Wu J., Wei L., Xia S., Zhu C., Yan J. (2023). Conductive Chromotropic Fiber Filament Sensors with Ultrahigh Stretchability for Wearable Sensing Textiles toward 3D Optical Motion Capture. J. Mater. Chem. A Mater. Energy Sustain..

[10] Linthorne N.P. (2001). Analysis of Standing Vertical Jumps Using a Force Platform. Am. J. Phys..

[11] Brooks E.R., Benson A.C., Bruce L.M. (2018). Novel Technologies Found to Be Valid and Reliable for the Measurement of Vertical Jump Height with Jump-and-Reach Testing. J. Strength Cond. Res..

[12] Lake J., Mundy P., Comfort P., McMahon J.J., Suchomel T.J., Carden P. (2018). Concurrent Validity of a Portable Force Plate Using Vertical Jump Force-Time Characteristics. J. Appl. Biomech..

[13] McErlain-Naylor S., King M., Pain M.T.G. (2014). Determinants of Countermovement Jump Performance: A Kinetic and Kinematic Analysis. J. Sports Sci..

[14] Wade L., Lichtwark G.A., Farris D.J. (2020). Comparisons of Laboratory-Based Methods to Calculate Jump Height and Improvements to the Field-Based Flight-Time Method. Scand. J. Med. Sci. Sports.

[15] McMahon J.J., Suchomel T.J., Lake J.P., Comfort P. (2018). Understanding the Key Phases of the Countermovement Jump Force-Time Curve. Strength Cond. J..

[16] Morin J.-B., Samozino P. (2016). Interpreting Power-Force-Velocity Profiles for Individualized and Specific Training. Int. J. Sports Physiol. Perform..

[17] Gonçalves C., Baptista R., Tufano J., Blazevich A.J., Vieira A. (2024). Error in Jump Height Estimation Using the Flight Time Method: Simulation of the Effect of Ankle Position between Takeoff and Landing. PeerJ.

[18] Silva M., Antunes H.D., Sousa A., Nakamura F.Y., Sampaio A.R., Pimenta R. (2025). Profiling of Physical Qualities of Highly Trained Portuguese Youth Soccer Players. Appl. Sci..

[19] Chang E., Norcross M.F., Johnson S.T., Kitagawa T., Hoffman M. (2015). Relationships between Explosive and Maximal Triple Extensor Muscle Performance and Vertical Jump Height. J. Strength Cond. Res..

[20] Pueo B., Lipinska P., Jiménez-Olmedo J.M., Zmijewski P., Hopkins W.G. (2017). Accuracy of Jump-Mat Systems for Measuring Jump Height. Int. J. Sports Physiol. Perform..

[21] Espitia-Mora L.A., Vélez-Guerrero M.A., Callejas-Cuervo M. (2024). Development of a Low-Cost Markerless Optical Motion Capture System for Gait Analysis and Anthropometric Parameter Quantification. Sensors.

[22] Fallahtafti F., Wurdeman S.R., Yentes J.M. (2021). Sampling Rate Influences the Regularity Analysis of Temporal Domain Measures of Walking More than Spatial Domain Measures. Gait Posture.

[23] Suo X., Tang W., Li Z. (2024). Motion Capture Technology in Sports Scenarios: A Survey. Sensors.

[24] Pueo B. (2016). High Speed Cameras for Motion Analysis in Sports Science. J. Hum. Sport Exerc..

[25] Miller E., Kaufman K., Kingsbury T., Wolf E., Wilken J., Wyatt M. (2016). Mechanical Testing for Three-Dimensional Motion Analysis Reliability. Gait Posture.

[26] Gabriel D.A., Kamen G., Frost G. (2006). Neural Adaptations to Resistive Exercise: Mechanisms and Recommendations for Training Practices. Sports Med..

[27] Markovic G., Mikulic P. (2010). Neuro-Musculoskeletal and Performance Adaptations to Lower-Extremity Plyometric Training. Sports Med..

[28] World Health Organization Be Healthy Iniciative: Physical Activity. https://www.who.int/initiatives/behealthy/physical-activity.

[29] Pimenta R., Lopes T., Bruno P., Veloso A. (2023). Effects of Repeated Sprints on Hamstring Active Shear Modulus Pattern and Neuromuscular Parameters in Football Players with and without Hamstring Strain Injury history—A Retrospective Study. Appl. Sci..

[30] Pimenta R., Correia J.P., Vaz J.R., Veloso A.P., Herzog W. (2024). Hamstrings Passive and Active Shear Modulus: Implications of Conventional Static Stretching and Warmup. J Sci Med Sport.

[31] Eythorsdottir I., Gløersen Ø., Rice H., Werkhausen A., Ettema G., Mentzoni F., Solberg P., Lindberg K., Paulsen G. (2024). The Battle of the Equations: A Systematic Review of Jump Height Calculations Using Force Platforms. Sports Med..

[32] Yamashita D., Murata M., Inaba Y. (2020). Effect of Landing Posture on Jump Height Calculated from Flight Time. Appl. Sci..

[33] Samozino P., Morin J.-B., Hintzy F., Belli A. (2010). Jumping Ability: A Theoretical Integrative Approach. J. Theor. Biol..

[34] de Blas X., Padullés J.M., López del Amo J.L., Guerra-Balic M. (2012). Creation and Validation of Chronojump-Boscosystem: A Free Tool to Measure Vertical Jumps. (Creación Y Validación de Chronojump-Boscosystem: Un Instrumento Libre Para La Medición de Saltos Verticales). Rev. Int. Cienc. Deporte.

[35] Bosco C., Luhtanen P., Komi P.V. (1983). A Simple Method for Measurement of Mechanical Power in Jumping. Eur. J. Appl. Physiol. Occup. Physiol..

[36] Cohen J. (2013). Statistical Power Analysis for the Behavioral Sciences.

[37] Sawilowsky S.S. (2009). New Effect Size Rules of Thumb. J. Mod. Appl. Stat. Methods.

